# Institutional pressures and green supply chain integration intention: Evidence from Chinese manufacturing firms

**DOI:** 10.1371/journal.pone.0322200

**Published:** 2025-05-07

**Authors:** Bochen Zhang, Shukuan Zhao, Dong Shao, Xueyuan Fan, Shuang Wang

**Affiliations:** 1 Business School, Yangzhou University, Yangzhou, China; 2 School of Management, Jilin University, Changchun, China; 3 Business School, Northeast Normal University, Changchun, China; University of Central Punjab, PAKISTAN

## Abstract

Green supply chain integration has become the key for manufacturing firms to cope with environmental challenges and gain sustainable competitiveness, but increasing the intention of firms to implement green supply chain integration is still a significant challenge. To respond to this issue, this study aims to theoretically discuss and empirically investigate the influence mechanism of institutional pressures on corporate green supply chain integration intention based on the theory of planned behavior. This study used a survey method to collect data from Chinese manufacturing firms, and the 292 finalized responses were analyzed using SPSS and AMOS. The findings indicate that institutional pressures, i.e., coercive and normative pressures, positively affect firms’ intention to implement green supply chain integration. The study also exposed the executives’ environmental awareness positively moderates the effect of coercive and normative pressures on green supply chain integration intention. Furthermore, executives’ self-efficacy positively moderates the effect of normative pressure on green supply chain integration intention. The findings of this study help deepen the understanding of the formation mechanism of green supply chain integration intention, which provides practical insights for effectively promoting green supply chain integration and realizing green transformation and high-quality development of manufacturing enterprises.

## 1. Introduction

With global warming and increasing environmental pollution, reducing carbon emissions and realizing sustainable development have become the focus of attention for all countries [1]. As an intrinsic driving force for economic growth, the manufacturing industry, with its inherent productive capacity and accumulation of knowledge, is the key to the sustained and high-quality development of a country’s economy and is also an essential support for constructing strategic advantages for future development [2]. However, in the process of rapid expansion of the manufacturing industry, the crude development model of “high input, high consumption, and high pollution” has brought about a large amount of energy consumption and pollutant emissions, which seriously threatens the sustainable development of the economy [3]. Therefore, there is an urgent need for manufacturing enterprises to reshape the new development model and transition to green development. Since the manufacturing industry typically encompasses a multitude of production steps and material transformation processes, the challenges to its development cannot be solved by the efforts of manufacturing firms alone [4]. Therefore, integrating supply chain partners into green transformation and constructing a sustainable new business model has become necessary for manufacturing enterprises to cope with challenges, break the deadlock, and open up new horizons [5]. In this context, green supply chain integration has emerged.

Green supply chain integration, as a new green supply chain strategy model, not only involves internal environmental management practices, but also extends the green concept to a series of supply chain management activities, including design, procurement, transportation, and end-processing of the product; this takes core manufacturing enterprises as the fulcrum, rely on the green cooperation between upstream suppliers and downstream customers, and promote the realization of green value co-creation among enterprises in the supply chain [6, 7]. Therefore, many excellent enterprises actively practice green supply chain integration as a realistic choice to enhance brand reputation and fulfill their social responsibility. For example, Huawei, BYD, Dell, and other companies actively promote collaboration with upstream and downstream supply chain partners to jointly build a “green supply chain,” which realizes a win-win situation regarding environmental and economic performance. However, although green supply chain integration has become a strategic choice for manufacturing enterprises to realize green transformation, the green supply chain integration practice of manufacturing enterprises still faces a series of practical challenges from the practical point of view. According to the Green Supply Chain CITI Index (2023) published by the Institute of Public and Environmental Affairs (IPE), many enterprises that have made environmental and climate commitments still fail to effectively implement green supply chain management practices, resulting in a low level of green supply chain management. This may be because implementing green supply chain management and integration strategies involves changes in internal business processes, resource allocation, and significant changes in external supply chain relationships [8]. Consequently, manufacturing enterprises usually lack the willingness and motivation to undertake green supply chain integration due to its characteristics of high investment and risk, which leads to the promotion of green supply chain integration practices in manufacturing enterprises facing many obstacles and the effect of green supply chain integration cannot be fully realized [9]. Therefore, how to promote the intention of manufacturing enterprises to implement green supply chain integration has become a critical issue that needs to be explored urgently.

According to the theory of planned behavior, institutional pressure is the external social pressure perceived by firms when deciding whether to engage in a specific behavior, and it is one of the crucial factors in determining their behavioral intentions [10]. Since firms’ behaviors are often embedded in specific institutional environments, firms’ behavioral intentions and strategic choices are inevitably constrained by the external institutional environment [11]. Therefore, further discussion is required on whether institutional pressures impact manufacturing firms’ intention to implement green supply chain integration. Additionally, the theory of planned behavior suggests that a firm’s behavioral intention is jointly influenced by subjective norms, behavioral attitudes, and perceived behavioral control [12, 13]. In other words, behavioral attitudes and perceived behavioral control will play an essential role in the relationship between subjective norms and behavioral intentions [14]. Since green supply chain integration is a complex green strategic practice that requires the active participation and support of corporate executives, the intention of manufacturing companies to implement green supply chain integration depends on executives’ perceptions and judgments [15]. Consequently, whether executives’ environmental awareness and self-efficacy can influence the relationship between institutional pressures and green supply chain integration intention (GSCII) is also one of the main concerns of this study.

In response to the significant strategic need for green transformation and development of manufacturing enterprises, this study aims to analyze the relationship between institutional pressures and GSCII, and identify the moderating effects of executives’ environmental awareness and self-efficacy. Consequently, this study seeks to make the following theoretical contributions. First, this study investigates the mechanism through which institutional pressure affects GSCII based on the theory of planned behavior (TPB), providing a new explanatory path for the motivation behind the formation of GSCII. Although existing literature has explored drivers of green supply chain integration, such as green entrepreneurial orientation [16], big data capabilities [17], and governance mechanisms [18], most studies focus on the behavioral outcomes of GSCII rather than its motivational antecedents. From the perspective of TPB, this paper examines the unique relationship between institutional pressure and GSCII, which not only addresses the gap in GSCII antecedent studies but also extends the applicability of TPB. Second, this study examines the moderating role of executives’ environmental awareness and self-efficacy in the relationship between institutional pressure and GSCII, offering new insights into the contextual mechanisms of institutional pressure. Previous studies on institutional pressures predominantly emphasizes their direct effects on corporate environmental behavior [19, 20], neglecting the significant role of corporate subjective initiative. This study innovatively incorporates executives’ environmental awareness and self-efficacy into the analytical framework and demonstrates the ‘joint effect’ of multi-level factors on GSCII, which clarifies the boundary conditions of the relationship between institutional pressure and GSCII and provides a scientific and practical foundation for improving the GSCII of manufacturing enterprises. Third, this study analyzes the driving mechanisms of GSCII in Chinese manufacturing enterprises, responding to scholars’ calls for research on China’s green supply chain management practices [8,21]. Most existing studies on green supply chain management practices focus on developed countries, and their conclusions are difficult to apply to enterprises’ green supply chain practices within China’s unique institutional environment. Through rigorous analysis of Chinese manufacturing samples, this study accurately identifies the institutional factors and managerial contextual characteristics that affect enterprises’ GSCII, thereby providing a specific pathway to enhance the GSCII of Chinese manufacturing enterprises.

## 2. Theory and hypothesis development

### 2.1 Theoretical foundations

The theory of planned behavior (TPB) is a critical theory that can systematically explain individuals’ general behavioral decision-making process, which American scholar Ajzen proposed based on the theory of rational behavior [13]. TPB suggests that an individual’s attitudes, perceived social norms, and perceived behavioral control influence their actual behavior and intention to engage in that behavior. Generally, when individuals perceive stronger subjective norms, more positive attitudes, and greater control over a specific behavior, they have a solid intention to perform it and are more likely to do so [22, 23]. TPB integrates a variety of factors, including the behavioral subject, the internal management of the organization, and the external environment of the organization, and provides a systematic framework for analyzing both the individual’s and the organization’s behavioral intentions, which has been widely applied in research across various fields such as management and sociology. In recent years, with the increasing severity of environmental problems, TPB has been proven effective in explaining enterprises’ green behavior and intention [24]. For example, Hong et al.(2022) [25] analyzed the drivers of Chinese enterprises adopting sustainable supply chain management based on TPB. They found that consumers’ environmental demands, executives’ support, and internal management capabilities of enterprises can significantly increase firms’ intention to adopt sustainable supply chain management practices. Shou et al.(2023) [26] analyzed the impact of subjective norms on enterprises’ green innovation in the supply chain. They found that injunctive norms and descriptive norms in the supply chain are the key driving factors in effectively promoting enterprises’ green innovation actions. Given this, TPB provides a persuasive framework for explaining the drivers of manufacturing firms’ intention to implement green supply chain integration.

Specifically, subjective norms refer to the social pressures that actors perceive when deciding whether to perform a specific behavior, reflecting the influence of significant groups on actors’ behavioral decisions [13]. Since firms’ behaviors are often embedded in specific institutional environments, external pressures inevitably influence their managerial decisions and behavioral intentions [11]. Established studies typically categorize institutional pressures into three dimensions: coercive, normative, and imitative [27]. Since the green supply chain integration practice has just started and has yet to form a noticeable imitation effect among enterprises, this study chooses coercive and normative pressures to represent subjective norms. Behavioral attitudes reflect the extent to which an actor enjoys or identifies with a specific behavior and their overall expected evaluation of that behavior [28]. Since green supply chain integration has a certain degree of risk and uncertainty, a change in the intrinsic values of enterprises is a prerequisite if they want to shift from the traditional supply chain model to the green supply chain model [21]. Regarding the attitude of manufacturing enterprises toward green supply chain integration behavior, the environmental awareness of executives may be an essential indicator. Executives’ environmental awareness refers to the level of executives’ attention, interpretation, judgment, and cognition of environmental issues and green development information when facing a complex external environment, i.e., whether executives are aware of the adverse impacts of the firms’ production and operation activities on the environment as well as the benefits that may be brought about by the implementation of green supply chain strategies [29, 30]. Therefore, this study chooses executives’ environmental awareness to represent behavioral attitudes. Perceived behavioral control refers to the degree to which an actor perceives it is easy or difficult to perform a specific behavior [14]. Established research has shown that self-efficacy reflects an individual’s perceived control over a specific behavior [31], i.e., an executive’s positive or negative evaluation of their ability to successfully organize and carry out green supply chain integration practices. Therefore, executives’ self-efficacy was chosen to represent perceived behavioral control in this study.

In summary, this study selects institutional pressures (i.e., coercive and normative pressures), executives’ environmental awareness, and self-efficacy as essential factors affecting GSCII to be explored under the TPB research framework.

### 2.2 Coercive pressure and GSCII

Coercive pressure is the pressure that perceives from the state, government, and other power institutions to monitor the organization’s behavior through enacting laws and regulations [32]. In recent years, China has faced higher production costs and severe environmental problems due to the overconsumption of natural resources [33]. To achieve the coordinated development of the economy and the environment, the Chinese government has implemented strict environmental protection measures to supervise and constrain the ecological behavior of enterprises [34]. With the escalation of environmental protection inspections, enterprises and their supply chains have been increasingly penalized for their polluting behaviors [10]. In this context, constructing a green supply chain and carrying out green supply chain integration practices are the keys for enterprises to gain legitimacy and ensure the smooth operation of the supply chain to realize sustainable development [16]. Previous studies have found that coercive pressure is essential for companies to implement green supply chain management [19]. For example, Qi et al.(2024)[35] found that the intention of enterprises to implement green supply chain management practices tends to be low due to the high cost and complex process of green supply chain strategy practices. In this context, the government’s strict environmental regulations can effectively stimulate the intention of enterprises to protect the environment and promote their green supply chain management practices. Wen et al.(2023) [15] point out that enterprises will actively adopt green supply chain management practices to avoid potential costs and legal liabilities arising from non-compliance to meet the government’s environmental policies and requirements. Thus, the coercive pressure from the promulgation and implementation of environmental regulations will significantly impact firms’ intention to implement green supply chain integration.

Specifically, strict environmental regulations not only increase the ecological governance costs of enterprises, but also strengthen the supervision and punishment of non-compliant enterprises [36]. If enterprises do not comply with environmental regulations, the government may take punitive measures against non-compliant enterprises, such as pollution fines, mandatory shutdowns, and revocation of the enterprise’s business licenses, which damages the resources owned by enterprises and threatens the survival and development of enterprises [37]. Therefore, enterprises will tend to implement green supply chain integration to avoid political risks and legal sanctions and safeguard their environmental legitimacy. Additionally, government environmental regulations challenge firms’ ecological behavior and require the entire supply chain wherein firms operate to demonstrate sustainability [38]. Due to the risk transmission effect of the supply chain, a violation in one link of the supply chain will lead to a cascading reaction in the entire supply chain. Once the enterprises in the supply chain are penalized for violating environmental standards, they will face the possibility of production stoppage or restriction, which increases the risk of disruption of the whole supply chain and causes enterprises to face severe economic losses [39, 40]. In this context, enterprises will tend to implement green supply chain integration to obtain environmental legitimacy and guarantee the stable operation of enterprises and even the whole supply chain. Based on the above analysis, this study proposes the following hypotheses:

Hypothesis 1 (H1): Coercive pressure positively affects firms’ GSCII.

### 2.3 Normative pressure and GSCII

Normative pressure is the pressure that drives firm-specific behaviors generated by social values and behavioral norms in an organization’s field [41]. Unlike coercive pressure, normative pressure regulates and guides organizational behavior through intangible rules such as moral beliefs, values, and social expectations [42]. Under normative pressure, firms usually align themselves with other members of the same institutional field regarding behavioral standards and norms to satisfy social expectations. Otherwise, firms will be subject to moral and ethical domination and thus lose business legitimacy [43]. With the increasing problems of environmental pollution, resource shortage, and ecological balance destruction, sustainable development has become a hot social concern [1]. Previous studies have found that the green strategic behavior of enterprises has been regarded as a key indicator of their legitimacy and reputation in the context of sustainable development [44]. Therefore, enterprises will actively promote green supply chain management practices to fulfill social expectations and ensure legitimacy and sustainable access to valuable resources [45]. Liu et al.(2023) [46] further confirm that stakeholders’ green expectations and aspirations are the key motivators to stimulate firms’ willingness to implement green supply chain management practices. Thus, this study suggests that normative pressure will significantly impact the intention of enterprises to implement green supply chain integration.

Specifically, the positive feedback and favorable brand image obtained by enterprises in social groups can deepen the relationship with stakeholders and lead to more resource allocation for enterprises [47]. This intrinsic value will strengthen firms’ recognition of taking environmental responsibility and make them realize the importance of implementing green supply chain strategies [43]. Consequently, the expectations of stakeholders in the organizational field for enterprises to implement green supply chain strategies will make enterprises regard green supply chain integration as a necessary way for them to achieve long-term development [9,47], which will increase the motivation of enterprises to choose a green supply chain strategy and stimulate their intention and motivation to implement green supply chain integration. Additionally, with the increasingly severe environmental problems, the media have launched heated debates and criticisms of enterprises that only pursue business interests and neglect sustainable development. With the wide dissemination of public opinion and media information, the normative pressure faced by enterprises increases [48]. If a company’s behavior goes against the collective intent or social norms, its unsocial behavior will be exposed to social controversy, making it the center of public opinion and even triggering negative public opinion about its supply chain [49]. Therefore, violating the normative requirements will lead to the exclusion of the entire supply chain and the loss of legitimacy, leading to severe economic losses and business risks [10]. Consequently, driven by the pursuit of “legitimacy” and “competitive advantage,” enterprises are more inclined to adopt green supply chain integration practices to improve their social image and gain more recognition and support. Based on the above analysis, this study proposes the following hypotheses:

Hypothesis 2 (H2): Normative pressure positively affects firms’ GSCII.

### 2.4. The moderating role of executives’ environmental awareness

In recent years, an increasing number of scholars have explored the reasons for enterprises to engage in green practices from the perspective of the external institutional environment and have argued that institutional pressures cause enterprises to pay attention to environmental issues, which leads to a homogeneous tendency of green behaviors [32]. However, scholars have found that the response of enterprises to institutional pressures is heterogeneous in the same institutional environment. In fact, firms’ responses to environmental issues are driven by both external pressures and internal dynamics [50]. Implementing green supply chain integration consumes a certain amount of corporate resources, and its benefits are hidden and long-term, leading to difficulties in identifying the potential benefits of green supply chain integration [51]. Consequently, even in the face of institutional pressures, the intention and motivation of enterprises to implement green supply chain integration are often insufficient. In this context, executives, as the key driving force within the firms, their awareness and attitudes towards environmental issues and green development concepts will directly affect the firms’ intention to implement green supply chain integration [52]. Thus, executives’ environmental awareness will play an essential moderating role in the relationship between institutional pressures and firms’ GSCII.

Executives’ environmental awareness is the perception of environmental issues formed by executives based on their knowledge structure and values, which reflects their attitude toward environmental issues and the concept of green development [29]. Studies have found that executives’ environmental awareness can affect the initiative of enterprises to implement environmental strategies, thus becoming an essential factor in determining whether institutional pressure can be effective [53]. On the one hand, executives with solid environmental awareness usually pay close attention to the latest requirements and directions of government environmental policies [54]. Therefore, a high degree of attention and sensitivity to environmental policies enable them to perceive the seriousness and urgency of environmental issues, as well as the threat of loss of legitimacy and the crisis of ecological protection risks under coercive pressure [55]. To avoid potential penalties and reputational damage and ensure the enterprise’s environmental legitimacy, executives with solid environmental awareness will tend to strictly comply with environmental regulations and reduce the negative impacts of the enterprise on the ecological environment through the implementation of green supply chain integration. In addition, executives with solid environmental awareness tend to have a higher sense of environmental responsibility and regard the coordinated development of the economy and the environment as an essential corporate responsibility and obligation [56]. Therefore, in the face of the government’s coercive pressure, executives with solid environmental awareness regard minimizing adverse impacts on the environment and achieving green development as an aspect of corporate responsibility, and thus, they are more willing to take active measures to respond to the government’s policy requirements [57], which further enhances the positive role of coercive pressure on enterprises’ intention to implement green supply chain integration.

On the other hand, executives with solid environmental awareness can promptly perceive environmental pressures from customers, industry associations, and other stakeholders and recognize the possible negative consequences of violating social norms, such as loss of organizational legitimacy and public opinion risks [56]. Therefore, under normative pressure, executives with solid environmental awareness will view the implementation of green strategies as a key way to reduce the negative impacts of the enterprise [58]. In addition, executives with solid environmental awareness tend to devote their limited attention to issues related to green development so they can obtain and grasp more green information promptly and thus keenly capture the green market opportunities embedded in normative pressure [32]. This enables them to clarify their enterprises’ future green development direction and respond to the normative pressure with a positive attitude [59]. To be consistent with the green development culture advocated by industry norms, increase stakeholders’ trust and support for the enterprise, and thus gain a sustainable green competitive advantage, executives with solid environmental awareness will be more inclined to send signals to the outside world that they are proactively fulfilling their social responsibility through the implementation of green supply chain strategies [60]. In this process, the intention of enterprises to implement green supply chain integration will be further stimulated, thus enhancing the positive effects of normative pressure. Based on the above analysis, this study proposes the following hypotheses:

Hypothesis 3a (H3a): Executives’ environmental awareness positively moderates the effect of coercive pressure on firms’ GSCII.

Hypothesis 3b (H3b): Executives’ environmental awareness positively moderates the effect of normative pressure on firms’ GSCII.

### 2.5. The moderating role of executives’ self-efficacy

Since green supply chain integration has high risk and uncertainty, enterprises are bound to evaluate and weigh whether they can successfully implement green supply chain integration before deciding whether to carry out green supply chain integration [17]. As the core of enterprise management, executives hold necessary strategic decision-making power in the enterprise [10]. If executives lack confidence in implementing green supply chain integration, it will be difficult for the enterprise to overcome the setbacks and failures of implementing green supply chain integration [61]. Therefore, the enterprise’s intention to carry out green supply chain integration will be affected by the degree of psychological readiness of the executives. According to TPB, self-efficacy as an individual’s overall perception of his ability, i.e., the subjective judgment of whether he believes he can carry out effective behaviors, is regarded as a trigger for the behavioral intention of a firm [31]. Therefore, executives’ self-efficacy is an essential factor influencing the firms’ GSCII and will play an important moderating role in the relationship between institutional pressures and GSCII.

Executives’ self-efficacy reflects the degree of confidence that executives have in their ability to perform green supply chain integration; the more significant the self-efficacy, the more confident they are in achieving desired goals and controlling uncertain environments [62]. Studies have found that green self-efficacy significantly increases managers’ willingness to protect the environment [63]. Therefore, managers with higher green self-efficacy are more likely to pay close attention to changes in the external environment and take green strategic measures to fulfill their corporate social responsibility [64], which significantly impacts the effectiveness of institutional pressures. Specifically, executives with high self-efficacy typically have higher internal control points, and they perceive that green supply chain integration activities are less complicated and that the challenges faced during the integration process are within their control [65]. Therefore, they have high confidence in accomplishing the tasks and goals of the green supply chain integration process, and they are confident in participating in green supply chain integration activities [62]. Thus, in the face of institutional pressures, their intention to choose green supply chain integration activities to meet social expectations and maintain legitimacy is higher. Additionally, due to institutional pressures provide opportunities to develop new markets and obtain various information resources, executives are more inclined to positively assess the potential benefits of implementing green supply chain integration when they believe that they are in control of the necessary resource conditions required for green supply chain integration practices and have better control over the progress and results of carrying out green supply chain integration [66]. In this case, executives do not worry about the negative impacts of institutional pressures but instead, see the challenges of institutional pressures as opportunities and are more likely to form favorable psychological expectations of green supply chain integration practices [67]. Moreover, as self-efficacy increases, executives’ risk-taking ability also increases, which will further motivate executives to show a stronger risk-taking orientation in the corporate decision-making process [68]. In this context, companies are more willing to try to create value by utilizing green supply chain integration practices, which helps stimulate the intention and motivation of companies to carry out green supply chain integration. On the contrary, executives with low self-efficacy have weaker psychological tolerance and risk-taking intentions and tend to focus on the consequences of failure in green supply chain integration [69, 70]. Therefore, even if the implementation of green supply chain integration can bring higher benefits to enterprises, considering the difficulties or obstacles they will face in the process of green supply chain integration, low self-efficacy executives may adopt negative behaviors such as suspending part of the production activities to satisfy the institutional requirements, which reduces the intention of enterprises to carry out green supply chain integration. Based on the above analysis, this study proposes the following hypotheses:

Hypothesis 4a (H4a): Executives’ self-efficacy positively moderates the effect of coercive pressure on firms’ GSCII.

Hypothesis 4b (H4b): Executives’ self-efficacy positively moderates the effect of normative pressure on firms’ GSCII.

**Fig 1:**
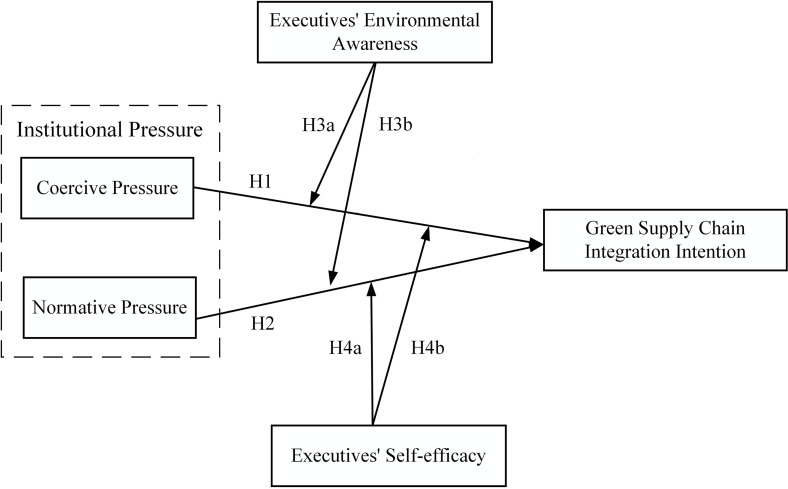
Conceptual model.

Based on the above analysis, a conceptual model is proposed for this study, as shown in Fig 1.

## 3. Methodology

### 3.1. Data collection

This study focuses on manufacturing enterprises in China, and there are several reasons for choosing China as the research background. Firstly, after more than 40 years of rapid development, China has become the second-largest economy in the world. During this evolution, the contribution of the manufacturing industry has been particularly prominent. Still, its crude economic growth model has brought about serious environmental problems and greatly hindered the high-quality development of the manufacturing industry [2]. As an essential part of the global supply chain, China’s manufacturing industry must take the initiative to shift to a green development model, thereby leading and driving more countries to achieve sustainable development [33]. Secondly, to address the challenges of global climate change and promote low-carbon development, China is actively building and improving the relevant institutional environment and policies to promote the green development of the manufacturing industry. From the state council’s “Guiding Opinions on Accelerating the Establishment of A Green and Low-Carbon Circular Economic Development System” to the “14th Five-Year Plan” for green industrial development and the policy documents of many ministries and commissions, all have proposed building a green supply chain and regard green supply chain management practice as an essential starting point for China’s industrial sector to implement the “double carbon” strategy. Therefore, this provides an ideal research background for this study to explore the impact of institutional pressures on manufacturing enterprises’ intentions to implement green supply chain integration.

This study adopts purposive and convenience sampling methods to collect survey data to explore the relationships between institutional pressure, executives’ environmental awareness, executives’ self-efficacy, and GSCII. Specifically, first, executives of Chinese manufacturing enterprises were chosen as the research subjects because they have a comprehensive understanding of enterprises’ green supply chain strategy practices, as well as profound insights into the enterprises’ green decision-making and strategic goals, which can help to ensure the quality of data collection. Second, according to the regional economic classification criteria of China’s National Bureau of Statistics, high-activity areas (Shanghai, Jiangsu, and Zhejiang), medium-activity areas (Henan and Shandong), and low-activity areas (Jilin and Liaoning) are selected as the research areas to ensure the diversity of sample selection and minimize the influence of economic development levels and geographical factors on the research results. Finally, the questionnaires were distributed in the selected regions through the following two channels: first, the members of the research team distributed the questionnaires through WeChat groups, enterprise mailboxes, and other online methods, leveraging their long-established social networks and contacts with manufacturing enterprises; second, the questionnaires were distributed and collected in cooperation with a professional management consulting firm. Prior to implementation, the management consulting company staff was thoroughly briefed on the main objectives of the study to ensure a full understanding of the study’s purpose, followed by a online questionnaire survey for the executives of manufacturing enterprises who met the research requirements.

In addition, before data collection, this study provided a “Participant Information and Consent Form” to potential participants and informed them of the academic purpose and intention of the study to ensure that they completed the questionnaire authentically. This formal research began in March 2022 and ended in May 2022; 450 questionnaires were collected, and after excluding the questionnaires with shorter completion time, answers with apparent regularity, and other problems, obtaining 292 valid questionnaires with a recovery efficiency rate of 64.89%. As shown in Table 1, from the distribution of the effective sample enterprises in terms of firm size, age, ownership, and industry types, the distribution of the sample enterprises’ characteristics is relatively diversified. There is no apparent extreme distribution, which is basically in line with the overall status of China’s manufacturing enterprises, indicating that the sample has good representativeness.

**Table 1:** Profiles of sample firms.

Characteristics of firms	Frequency (N)	Percent (%)
Industry		
Food products	51	17.47
Communication and computer‐related equipment	70	23.97
Pharmaceutical and medical	33	11.30
General equipment	32	10.96
Electrical machinery and equipment	36	12.33
Chemical products and petrochemical industry	7	2.40
Transport equipment	10	3.42
Textiles and apparel	33	11.30
Others	20	6.85
Firm age		
≤ 5	2	0.68
6–10	71	24.32
11–15	106	36.30
≥16	113	38.70
Number of employees		
≤100	25	8.56
101–300	99	33.90
301–500	64	21.92
501–1000	54	18.49
1001–2000	19	6.51
>2000	31	10.62
Ownership structure		
Private enterprises	47	16.09
State‐owned enterprises	209	71.58
Joint venture	19	6.51
Foreign‐invested enterprises	7	2.40
Others	10	3.42
Total	292	

### 3.2. Measures

To measure the five research variables (coercive pressure, normative pressure, executives’ environmental awareness, executives’ self-efficacy, and GSCII), this study used validated scales from existing literature, with appropriate modifications based on the research context to ensure the applicability and accuracy of the scales. Specifically, institutional pressure was divided into two dimensions: coercive and normative pressure. The measurement of coercive pressure adopted from the research by Dai et al.(2021)[10] and Jiang et al.(2024)[53], comprising four measurement items. The measurement of normative pressure adopted from the research by Dai et al.(2021)[10], Geng and Dai(2024)[45], and Wang et al.(2018)[32], comprising four measurement items. The measurement of executives’ environmental awareness adopted from the research by Cao et al.(2022)[29], Tang et al.(2024)[56], and Yin et al.(2019)[54], comprising five measurement items, which reflect executives’ concerns, interpretations, judgments, and perceptions of environmental issues and green development from the perspectives of opportunistic environmental awareness and responsible environmental awareness. The measurement of executives’ self-efficacy adopted from the research by Chen et al.(2014)[69] and Zhang et al.(2023)[71], comprising five measurement items that reflect executives’ positive or negative evaluations of their ability to organize and implement green supply chain integration successfully. The measurement of GSCII adopted from the research by Jum’a et al.(2022)[72] and Singh and Joshi(2024)[20], comprising five measurement items, which assess an enterprise’s proactiveness, motivation, and effort in green supply chain integration. After determining the measurement indicators, this study invited three Ph.Ds in management to translate and proofread the established scale using the “translation-back-translation” method. The initial draft of the questionnaire was developed after review and revision by two experts in the field. Subsequently, to further enhance the validity and comprehensibility of the questionnaire, a pre-survey was conducted, and refinements were made to specific wording based on the feedback received, ultimately finalizing the complete questionnaire. All constructs were measured using a 7-point Likert scale, with “1” representing total disagreement and “7” representing total agreement (The questionnaire items are shown in the Supporting Information S2 File).

Additionally, to reduce the interference of enterprise characteristic factors on the results of this study and to improve the reliability of the results, this study chooses firm age, size, ownership, and industry type as control variables. Specifically, firm age is divided into four levels according to the establishment time of the enterprise; firm size is divided into five levels according to the number of employees; firm ownership is a dummy variable divided into state-owned enterprises and non-state-owned enterprises, with the values of 1 and 0; firm industry type is a dummy variable divided into high-pollution and non-high-pollution industries, with the values of 1 and 0.

## 4. Results

### 4.1. Common method variance

Since all the variables in this study were researched using the same questionnaire and filled by the same subjects, there may be a problem of common method variance [73]. Therefore, after all data collection, this study used Harman’s single-factor test to test the research data for common method variance. The results showed that the first factor explained 28.606% of the total variance without rotation, which is less than the critical value of 40%, indicating no serious common method variance in this study.

### 4.2. Reliability and validity

In this study, the reliability and validity of the questionnaire were tested using SPSS 25.0 and AMOS 24.0, and the results are shown in Table 2. In terms of reliability, this study used Cronbach’s α to test the consistency of the variable items, and the results showed that Cronbach’s α value of each measurement variable is greater than 0.7, indicating that the scales have good internal consistency and reliability. In terms of validity, the scales used in this study are all well-established scales that are highly relevant to the topic of this study and have been cited repeatedly. Hence, the scales possess good content validity. Subsequently, a validated factor analysis of the variables is conducted using AMOS 24.0. The results showed that each measurement variable’s standardized factor loading values are greater than 0.5, the CR values are greater than 0.7, and the AVE values are greater than 0.5, indicating that the scale has good convergent validity. Additionally, the absolute fit index χ2/df value is 1.066 (<3), the RMSEA value is 0.015 (<0.05), the GFI value is 0.936, and the relative fit indexes NFI, IFI, and CFI values are 0.937, 0.996, and 0.996, respectively, which are greater than 0.9. The above results were within a reasonable range, indicating a good ﬁt of the model. Moreover, as shown in Table 3, the square root of the AVE values of all latent variables is greater than the correlation coefficient between the variable and other latent variables, indicating that the scale has good discriminant validity. In conclusion, the reliability and validity test indicate an excellent choice of measurements for this study.

**Table 2:** Confirmatory factor analysis results.

Construct	Item	Factor loading	AVE	Cronbach’s α	CR
Coercive Pressure(CP)	CP1	0.759	0.599	0.853	0.856
	CP2	0.662			
	CP3	0.810			
	CP4	0.852			
Normative Pressure(NP)	NP1	0.807	0.648	0.879	0.880
	NP2	0.854			
	NP3	0.724			
	NP4	0.830			
Executives’ Environmental Awareness(EA)	EA1	0.847	0.543	0.852	0.855
	EA2	0.765			
	EA3	0.695			
	EA4	0.663			
	EA5	0.701			
Executives’ Self-Efficacy(SE)	SE1	0.819	0.662	0.906	0.907
	SE2	0.813			
	SE3	0.732			
	SE4	0.884			
	SE5	0.812			
Green Supply Chain Integration Intention(GSCII)	GSCII1	0.745	0.543	0.854	0.855
	GSCII2	0.822			
	GSCII3	0.735			
	GSCII4	0.689			
	GSCII5	0.685			

Abbreviation: AVE, average variance extracted

**Table 3:** Mean, standard deviations, and correlations of the constructs.

Constructs	Mean	SD	Skewness	Kurtosis	1	2	3	4	5	6	7	8	9
1. Firm Age	3.130	0.802	‒0.320	‒1.149	1								
2. Firm Size	3.123	1.440	0.603	‒0.539	0.358^**^	1							
3. Firm Ownership	0.158	0.365	1.890	1.582	0.047	0.153^**^	1						
4. Industry Type	0.425	0.495	0.306	1.919	‒0.079	‒0.093	‒0.143^*^	1					
5. CP	5.045	1.154	‒0.566	‒0.225	‒0.077	‒0.027	0.016	0.010	**0.774**				
6. NP	4.996	1.260	‒0.586	‒0.315	0.050	0.045	‒0.019	‒0.025	0.504^**^	**0.805**			
7. EA	4.804	1.225	‒1.127	1.202	0.086	0.142^*^	‒0.058	0.046	0.026	0.126^*^	**0.737**		
8. SE	4.828	1.408	‒0.820	‒0.114	0.069	0.117^*^	0.025	0.023	‒0.002	0.221^**^	0.441^**^	**0.814**	
9. GSCII	4.960	1.203	‒1.185	1.478	‒0.017	0.054	‒0.030	‒0.003	0.539^**^	0.443^**^	0.252^**^	0.151^**^	**0.737**

Note: The bold diagonal line indicates average variance‐extracted square roots.

N=292;

*p < 0.05,

**p < 0.01.

### 4.3. Descriptive statistics and correlation analysis

In this study, we conducted a detailed analysis of each variable’s means, standard deviations, and correlation coefficients using SPSS 25.0 software, with the results presented in Table 3. The descriptive statistics in Table 3 indicate that all variables’ means and standard deviations fall within acceptable ranges. Additionally, the absolute values of the correlation coefficients between the variables are less than 0.7, and the VIF values are much less than the critical value of 10, indicating that the regression model does not have serious multicollinearity problems. Consistent with prior studies, the skewness and kurtosis parameters were below 3.0 and 10.0, respectively [74, 75], indicating that the data approximates a normal distribution and meets the assumptions required for regression analysis. Furthermore, there are significant positive correlations between coercive pressure (β=0.539, p<0.01), normative pressure (β=0.443, p< 0.01), executives’ environmental awareness (β= 0.252, p<0.01), executives’ self-efficacy (β=0.151, p<0.01) and GSCII, and these results provide a preliminary verification of the hypotheses.

### 4.4 Hypothesis testing

In this study, the hypotheses were tested by hierarchical regression analysis. The data were standardized before hypothesis testing, and the VIF results for each model during the test were less than 2, indicating no multicollinearity problem. In addition, although this study proposed six hypotheses based on theoretical foundation and existing literature, it still cannot exclude that factors such as control variables may cause the test results to be inconsistent with the expected direction. To comprehensively and objectively assess the effects of independent and moderating variables, this study used SPSS 25.0 and a two-tailed test for regression analysis to improve the study’s rigor and reliability. The specific model settings and regression analysis results are shown in Tables 4 and 5 and Fig 5.

**Table 4:** Main effect regression analysis results.

Variables	Model 1	Model 2	Model 3
Firm age	‒0.043	‒0.001	‒0.059
Firm size	0.074	0.075	0.059
Firm Ownership	‒0.040	‒0.051	‒0.027
Industry type	‒0.005	‒0.008	0.005
CP		0.542^***^	
NP			0.443^***^
F value	0.427	24.222^***^	14.384^***^
R^2^	0.006	0.297	0.201
Adjusted R^2^	‒0.008	0.285	0.187

Note. N=292;

*p < 0.05,

**p < 0.01;

***p < 0.001.

**Table 5:** Moderating effect regression analysis results.

Variables	Model 4	Model 5	Model 6	Model 7	Model 8	Model 9	Model 10	Model 11
Firm age	-0.011	-0.018	-0.067	-0.060	-0.006	-0.003	-0.061	-0.013
Firm size	0.042	0.018	0.032	0.035	0.060	0.060	0.054	0.049
Firm ownership	-0.033	-0.035	-0.012	-0.028	-0.052	-0.052	-0.028	-0.023
Industry type	-0.020	-0.015	-0.006	0.011	-0.014	-0.011	0.003	0.011
CP	0.534^***^	0.551^***^			0.541^***^	0.541^***^		
NP			0.419^***^	0.457^***^			0.431^***^	0.541^***^
EA	0.232^***^	0.232^***^	0.200^***^	0.151^**^				
SE					0.148^**^	0.143^**^	0.055	0.104^*^
CP×EA		0.230^***^						
NP×EA				0.280^***^				
CP×SE						0.026		
NP×SE								0.385^***^
F value	25.506^***^	27.157^***^	14.919^***^	18.566^***^	22.244^***^	19.054^***^	12.153^***^	20.187^***^
R^2^	0.349	0.401	0.239	0.314	0.319	0.320	0.204	0.332
Adjusted R^2^	0.336	0.386	0.223	0.297	0.305	0.303	0.187	0.316

Note. N=292;

*p < 0.05,

**p < 0.01;

***p < 0.001.

**Fig 2:**
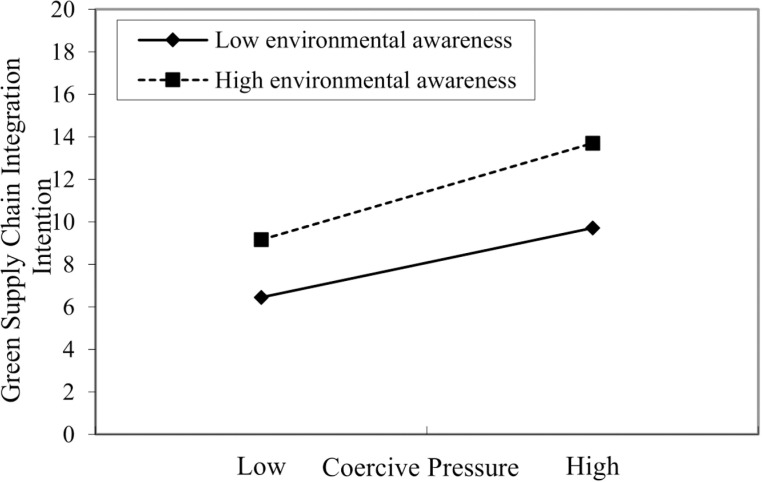
Interaction of coercive pressure and executives’ environmental awareness on GSCII.

**Fig 3:**
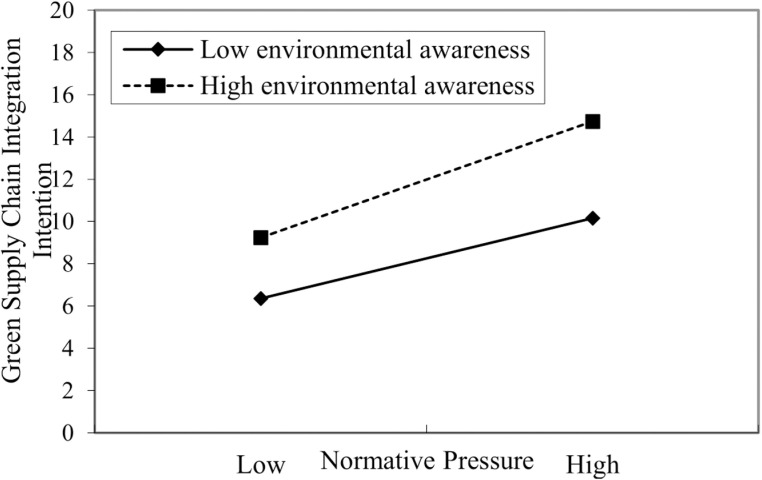
Interaction of normative pressure and executives’ environmental awareness on GSCII.

**Fig 4:**
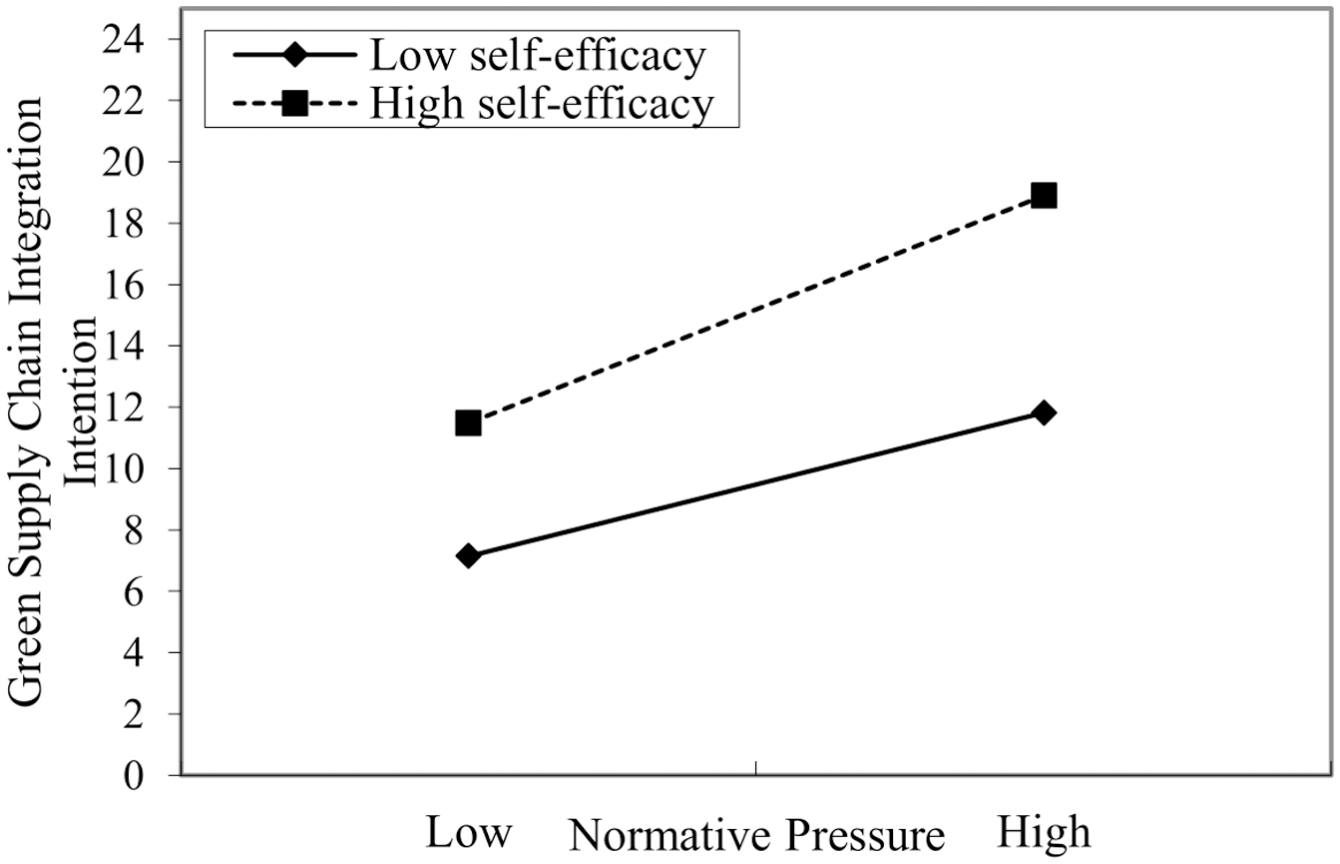
Interaction of normative pressure and executives’ self-efficacy on GSCII.

**Fig 5:**
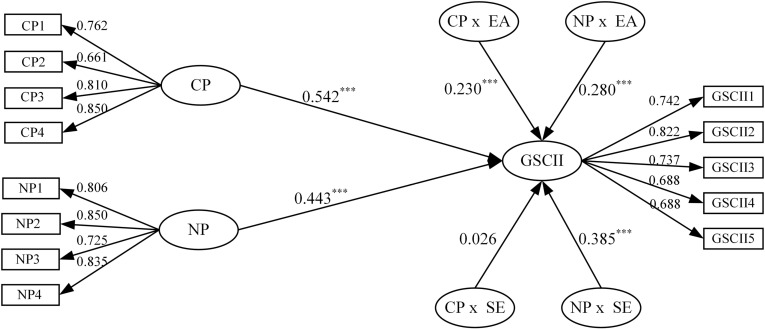
Effects of the regression model.

#### 4.4.1. Main effect test.

To verify the impact of the different institutional pressures (i.e., coercive and normative pressures) on the intention of enterprises to implement green supply chain integration, this study sequentially constructs Models 1–3 to test the above research hypotheses, and the regression results are shown in Table 4. In Table 4, Model 1 is the baseline model with only control variables, and Models 2 and 3 add coercive and normative pressures, respectively, based on Model 1. The results show that both coercive pressure (β=0.542, p<0.001) and normative pressure (β=0.443, p<0.001) positively affect GSCII. Meanwhile, compared to Model 1, the R^2^ of Models 2 and 3 increased, and the F-value reached a significant level. In summary, it shows that coercive and normative pressures positively affect GSCII, and hypotheses H1 and H2 are supported.

#### 4.4.2. Moderating effect test.

To avoid the problem of multicollinearity caused by the inclusion of interaction terms, this paper centered the independent variables (coercive and normative pressures) and moderating variables (executives’ environmental awareness and self-efficacy) and then brought the interaction terms of the independent variables and the moderating variables into the regression equation.

(1) The moderating role of executives’ environmental awareness

To verify the moderating effect of executives’ environmental awareness on the relationship between institutional pressures (i.e., coercive and normative pressures) and GSCII, this study sequentially constructs Models 4–7 to test the above research hypotheses, and the test results are shown in Table 5. In Table 5, Models 4 and 5 were used to test the moderating effect of executives’ environmental awareness on the relationship between coercive pressure and GSCII. Model 5 shows that the interaction term of coercive pressure and executives’ environmental awareness positively affect GSCII (β=0.230, p<0.001). Meanwhile, compared to Model 4, the R^2^ of Model 5 increased, and the F-value reached a significant level. In summary, it shows that executives’ environmental awareness positively moderates the relationship between coercive pressure and GSCII, and hypothesis H3a is supported.

In Table 5, Models 6 and 7 were used to test the moderating effect of executives’ environmental awareness on the relationship between normative pressure and GSCII. Model 7 shows that the interaction term of normative pressure and executives’ environmental awareness positively affect GSCII (β=0.280, p<0.001). Meanwhile, compared to Model 6, the R^2^ of Model 7 increased, and the F-value reached a significant level. In summary, it shows that executives’ environmental awareness positively moderates the relationship between normative pressure and GSCII, and hypothesis H3b is supported.

In addition, to more intuitively reflect the moderating effect of executives’ environmental awareness on the relationship between institutional pressures and GSCII, this study categorizes the awareness into high and low levels by adjusting the mean value by one standard deviation. The diagrams illustrate this moderating effect (see Figs 2 and 3). As shown in Figs 2 and 3, the impact of coercive and normative pressures on GSCII is more significant than when environmental awareness is low. Consequently, hypotheses H3a and H3b are further supported.

(2) The moderating role of executives’ self-efficacy

To verify the moderating effect of executives’ self-efficacy on the relationship between institutional pressures (i.e., coercive and normative pressures) and GSCII. This study sequentially constructed Models 8–11 to test the above research hypotheses, and the test results are shown in Table 5. In Table 5, Models 8 and 9 were used to test the moderating effect of executives’ self-efficacy on the relationship between coercive pressure and GSCII. Model 9 shows that the interaction term of coercive pressure and executives’ self-efficacy has no significant effect on GSCII (β=0.026, p>0.1). This suggests that the moderating role of executives’ self-efficacy between coercive pressure and GSCII is insignificant, and hypothesis H4a is not supported.

In Table 5, Models 10 and 11 were used to test the moderating role of executives’ self-efficacy on the relationship between normative pressure and GSCII. Model 11 shows that the interaction term of normative pressure and executives’ self-efficacy positively affects GSCII (β=0.385, p<0.001). Meanwhile, compared to Model 10, the R^2^ of Model 11 increased, and the F-value reached a significant level. In summary, it shows that executives’ self-efficacy positively moderates the relationship between normative pressure and GSCII, and hypothesis H4b is supported.

In addition, to more intuitively reflect the moderating effect of executives’ self-efficacy on the relationship between normative pressure and GSCII, this study categorizes the self-efficacy into high and low levels by adjusting the mean value by one standard deviation. The diagram illustrates this moderating effect (see Fig 4). As shown in Fig 4, the impact of normative pressure on GSCII is more significant than when self-efficacy is low. Consequently, hypothesis H4b is further supported.

## 5. Conclusions and discussion

### 5.1. Conclusions

In the context of globalization and sustainable development, manufacturing enterprises face both market and ecological challenges: identifying and adopting management models with competitive advantages in the market and implementing green practices that satisfy ecological balance [2]. Therefore, developing and implementing green supply chain integration has become essential for manufacturing enterprises to cope with environmental challenges and gain sustainable competitiveness [76]. However, since green supply chain integration is characterized by high investment and risk, most manufacturing enterprises are unwilling to implement green supply chain integration, resulting in the implementation of green supply chain integration not playing its proper role [9]. Therefore, improving the intention of manufacturing enterprises to implement green supply chain integration has become an important issue that needs to be solved. In this context, based on the theory of planned behavior, this study constructs a research framework with institutional pressures (i.e., coercive and normative pressures) as the independent variable, GSCII as the dependent variable, and executives’ environmental awareness and self-efficacy as the moderating variables. Subsequently, this study utilizes sample data from Chinese manufacturing enterprises to test the framework, and the following conclusions are mainly drawn.

First, institutional pressure is an essential factor influencing the intention of manufacturing enterprises to implement green supply chain integration, which can stimulate the intention and motivation of manufacturing enterprises to carry out green supply chain integration through two institutional isomorphisms: coercive and normative pressures. This finding corroborates the consensus of existing studies on the influential role of institutional pressure as an essential driving force to promote enterprises’ participation in green supply chain management practices [10,77]. On the one hand, the impact of coercive pressure on the behavioral decisions of manufacturing enterprises cannot be ignored in the context of China’s increasingly improved legal system of environmental protection [78]. Strict environmental regulations not only increase the ecological governance costs of enterprises [36], but also increase the punishment of non-compliant enterprises [37]. Therefore, manufacturing firms will actively engage in green supply chain integration to maintain legitimacy and signal a positive environmental posture to the outside world [79]. On the other hand, with the increasingly severe ecological and environmental problems, environmental protection and sustainable development have attracted widespread attention from society, and the informal institutional pressures faced by manufacturing firms is also increasing [80]. Under normative pressure, if manufacturing firms actively fulfill the rules and requirements of stakeholders, they can maintain the legitimacy of their existence and gain additional social resources and reputation [81]; if manufacturing firms do not comply with the social norms, it will lead to severe losses and risks for the firms and even for the whole supply chain [10]. Therefore, under the effect of “legitimacy” and “competitive advantage,” enterprises will tend to implement green supply chain integration to fulfill the social expectations of stakeholders, thus enhancing their legitimacy and improving their survivability.

Second, executives’ environmental awareness positively moderates the role of two dimensions of institutional pressures affecting GSCII. This suggests that executives’ environmental awareness affects their identification and interpretation of external institutional pressures to a certain extent, and this difference will be further projected into firms’ behavioral decisions. Therefore, executives’ environmental awareness will have a significant impact on the process effects exerted by institutional pressures [55]. Specifically, executives with high environmental awareness typically devote their attention to issues related to green development. Therefore, they can promptly perceive the threat of legitimacy loss and identify potential green market opportunities under institutional pressures [58]. In this context, such executives will more actively incorporate green supply chain integration into the strategic objectives to ensure the survival and development of the enterprise. Thus, the effect of two dimensions of institutional pressures (i.e., coercive and normative pressures) on the intention of enterprises to implement green supply chain integration will be further enhanced.

Third, unlike what was hypothesized in the hypotheses, the findings of this study confirm that whether executives’ self-efficacy plays a moderating role depends on the type of institutional pressures. Specifically, this study finds that executives’ self-efficacy positively moderates the process of normative pressure affecting GSCII. When executives have high self-efficacy, they are less worried about the risks and challenges of normative pressures and are more willing to utilize their capabilities to fulfill the demands of normative pressures and obtain more valuable resources for the firm [62,68]. Consequently, executives with greater self-efficacy will be more confident in meeting the normative pressure, which will further stimulate the intention of enterprises to carry out green supply chain integration. However, unlike what was hypothesized in the hypotheses, the empirical results of this study indicate that executives’ self-efficacy cannot play a moderating role between coercive pressure and GSCII. This may be because power organizations like the government still control the necessary resources for firms’ survival and development in the Chinese institutional context [82]. The coercive pressure from the government’s strict environmental regulations may lead to financial penalties and sanctions for manufacturing firms. To some extent, its adverse effects have a more far-reaching impact than the normative pressure [83]. Therefore, under coercive pressure, regardless of whether executives’ confidence and sense of control in carrying out green supply chain integration is at a high level, for the sake of survival and growth, executives will tend to respond positively to the government’s environmental regulation by implementing green supply chain integration based on the consideration of corporate interests and future development.

### 5.2. Theoretical contributions

The research in this study has the following theoretical contributions. First, this study deeply explores the mechanism of institutional pressures on GSCII based on the TPB, which not only advances the antecedent research on GSCII, but also expands the application scope of the TPB. Green supply chain integration is an essential way for manufacturing enterprises to realize green development, and it has become an important research topic. In the antecedent studies of green supply chain integration, scholars have explored the influence of factors on green supply chain integration based on different perspectives, such as green entrepreneurial orientation [16], big data capabilities [17], and governance mechanisms [18], which has accumulated rich research results in the field of green supply chain integration. However, the existing literature mainly focuses on the influence of various factors on the final green supply chain integration behavior and results, and few scholars focus on the generation of intention before behavior, which results in the motivation for the formation of GSCII is still unclear. Given this, this paper provides an exploratory explanation of this interesting relationship between institutional pressures and GSCII based on the TPB, which not only complements the antecedent research on GSCII and enriches the research framework of green supply chain integration, but also extends the application of the TPB in the field of research on corporate green supply chain management practices.

Second, this study incorporates executives’ environmental awareness and self-efficacy as moderating variables into the analytical framework, comprehensively analyzes the adaptive conditions of institutional pressures, which provides new theoretical explanations for the situational mechanisms affecting the effectiveness of institutional pressures, and expands the contextual path of research on GSCII. Most studies on institutional pressures focus on the direct impact of institutional pressure on the environmental behavior of enterprises [19, 20], usually ignoring the influence of the subjective initiative of the object, resulting in the failure of existing studies to adequately guide the green practice of manufacturing enterprises. Based on the TPB, this paper innovatively integrates institutional pressures, executives’ environmental awareness, self-efficacy, and GSCII into the same research framework and systematically presents the “joint effect” of multi-level factors on GSCII, which not only identifies the boundary conditions of the relationship between institutional pressures and GSCII, but also constructs a relatively complete analysis framework of driving factors of GSCII, which provides scientific decision-making basis for effectively improving GSCII of manufacturing enterprises.

Third, based on the real needs of China’s high-quality economic development and the development status quo of manufacturing enterprises, this study provides an in-depth analysis of the driving mechanism of manufacturing enterprises’ GSCII, thus responding to scholars’ calls for research on China’s green supply chain management practices [8,21]. Although the research on green supply chain management practices has attracted much attention from scholars, most studies have focused on developed countries, and relatively little attention has been paid to developing countries such as China. Unlike Western management practices, China’s unique institutional environment plays a vital role in the process of corporate strategy formulation, leading to behavioral tendencies that present unique characteristics different from those of Western enterprises. Thus, there is an urgent need for an in-depth exploration of green supply chain management practices in Chinese enterprises. Therefore, based on the Chinese background of comprehensively promoting green transformation, this study systematically identifies the institutional factors and management contextual features that affect the GSCII of manufacturing firms. The research results of this study provide specific path suggestions for effectively improving the GSCII of manufacturing firms and provide theoretical insights for China manufacturing enterprises to realize green development, which has significant theoretical value and practical significance.

### 5.3. Managerial insights

This paper provides an exploratory explanation of this interesting relationship between institutional pressures and GSCII, and the results of the study not only can effectively improve the GSCII of manufacturing enterprises to bring practical inspiration, but also fit the current situation of the government in promoting green development at the macro level, which provides specific theoretical references and suggestions for the government to formulate effective policies, and is of strong practical guidance significance. First, the government should further strengthen institutional construction and institutional isomorphism for manufacturing enterprises to enhance their GSCII. The research findings indicate that the different institutional pressures (i.e., coercive and normative pressures) are crucial for increasing the intention of manufacturing firms to implement green supply chain integration. Therefore, the Chinese government should play a better macro-control role, strengthen policy supervision and planning guidance, and form a linkage effect of coercive and normative pressures and a multi-purpose environmental governance system. On the one hand, the Chinese government should continue to strengthen the formulation and implementation of environment-related laws and regulations, continuously improve the policy system of environmental governance, strengthen the supervision and constraints on manufacturing enterprises by exerting its “coercive,” enhance the environmental awareness of manufacturing enterprises, and improve the intention of manufacturing enterprises to implement green supply chain integration. On the other hand, the Chinese government should strengthen the construction of ecological morality, build a green consumption environment, and further enhance environmental awareness in all fields of society. At the same time, it should focus on cultivating a green public opinion atmosphere, guiding the news media and stakeholders to play a good role in social supervision, increasing the reporting and disclosure of environmental pollution incidents, and better exerting its external governance function to improve the enthusiasm of manufacturing enterprises to carry out green supply chain integration.

Second, enterprises and governments should focus on cultivating and improving corporate executives’ environmental awareness and self-efficacy and fully play the corporate initiative’s positive role. The research findings show that executives’ environmental awareness and self-efficacy can modulate the relationship between institutional pressures and GSCII, and therefore, manufacturing enterprises should improve their intention and enthusiasm for green supply chain integration by “internal and external cultivation.” At present, the strict environmental policies and social supervision have made many manufacturing enterprises pay attention to the importance of “external cultivation,” but “internal cultivation” is also essential in improving the intention of manufacturing enterprises to implement green supply chain integration. On the one hand, China’s policy-makers should establish a relationship with corporate executives by organizing training courses for entrepreneurs, industry associations, and media campaigns to deliver environmental protection information to executives regularly to enhance their knowledge of environmental protection and to promote the establishment of executives’ awareness of environmental protection. At the same time, the government should also create a favorable green atmosphere, giving executives sufficient spiritual and material incentives. Therefore, executives can obtain high social recognition and support and stimulate their sense of self-efficacy. On the other hand, as essential decision-makers of corporate strategy, executives should pay close attention to domestic and international environmental policies and market trends in green demand, continuously environmental awareness, actively fulfill their social responsibility, and actively lead the enterprises to implement green supply chain integration strategy. At the same time, executives should also be good at self-empowerment and self-motivation, which should be transformed into internal confidence and fighting spirit to overcome the psychological barriers to the implementation of green supply chain integration and provide strong support for enhancing the motivation of manufacturing enterprises to carry out green supply chain integration.

### 5.4. Limitations and future research directions

There are certain limitations in this study that need to be further improved in future research. First, in terms of the research object, this study is conducted in the context of developing countries with special institutional environments, which may result in the findings not applying to other developing or developed countries. Future research can use more cross-country sample data to investigate the relationship between institutional pressures and firms’ GSCII and compare whether the differences between countries affect the relationship. Second, in terms of data collection, this study adopts static cross-sectional data, which cannot reflect the dynamic impact of institutional pressures on firms’ GSCII. Future research can utilize time series data or adopt case study methodology to track the dynamic changes in the effects of institutional pressures to obtain conclusions with more substantial explanatory power and robustness. Third, in terms of research content, this study examined the impact of coercive and normative pressures on firms’ GSCII respectively, but it has not yet explored whether these two pressures are complementary and whether their impact on firms’ GSCII has a combined effect, which is worth further exploring in future research.

## Supporting information

S1 Data Regression data. (XLSX)

S2 File List of scale items. (DOCX)

S3 Fig1 Conceptual model. XXX.
(TIF)

S4 Fig2 Interaction of coercive pressure and executives' environmental awareness on GSCII. (TIF)

S5 Fig3 Interaction of normative pressure and executives' environmental awareness on GSCII. (TIF)

S6 Fig4 Interaction of normative pressure and executives' self-efficacy on GSCII. (TIF)

S7 Fig5 Effects of the regression model. (TIF)
