# Supplementary material for: Institutional pressures and green supply chain integration intention: Evidence from Chinese manufacturing firms
Source: PLoS One. 2025 May 7;20(5):e0322200. doi: 10.1371/journal.pone.0322200 (PMC12058135; doi:10.1371/journal.pone.0322200)
Supplement: S2 File — (DOCX) [file pone.0322200.s002.docx]

**Appendix**

# List of scale items

**Coercive Pressure (CP)**

CP1: The government imposes severe penalties for corporate behavior that damages the environment. (Dai et al.,2021[10])

CP2: The government has established a series of strict environmental laws and regulations. (Dai et al.,2021[10])

CP3: The government publicizes the concept of green development in society through various channels. (Jiang et al.,2024[50])

CP4: The government promptly addresses public reports of corporate behavior that damages the environment. (Dai et al.,2021[10])

**Normative Pressure (NP)**

NP1: The industry association encourages the enterprise's green transformation and development. (Geng and Dai,2024[42])

NP2: Customers expect the enterprise to demonstrate environmental responsibility. (Dai et al.,2021[10])

NP3: Undertaking environmental responsibility is an essential requirement for an enterprise to enter this industry's market. (Wang et al.,2018[27])

NP4: If an enterprise's behavior is detrimental to the ecological environment, stakeholders will not support its survival and development. (Wang et al.,2018[27])

**Executives' Environmental Awareness (EA)**

EA1: Enterprises should consciously comply with the government's environmental policies. (Yin et al.,2019[51])

EA2: Enterprises should actively fulfill their social responsibility to protect the environment. (Tang et al.,2024[53])

EA3: I place great emphasis on the negative impacts of the enterprise's production and operational activities on the environment. (Yin et al.,2019[51])

EA4: A green supply chain integration strategy can enhance the corporate image.(Cao et al.,2022[24])

EA5: A green supply chain integration strategie can enhance an enterprise's overall competitiveness. (Cao et al.,2022[24])

**Executives' Self-Efficacy (SE)**

SE1: I can facilitate the implementation of corporate green supply chain integration practices. (Chen et al.,2014[67])

SE2: I am capable of addressing and overcoming the challenges associated with the implementation of green supply chain integration. (Chen et al.,2014[67])

SE3: I can coordinate with various departments and stakeholders to advance the implementation of green supply chain integration. (Zhang et al.,2023[69])

SE4: I can mobilize the necessary resources to fulfill the requirements of green supply chain integration. (Zhang et al.,2023[69])

SE5: I possess the managerial capabilities required for implementing green supply chain integration. (Zhang et al.,2023[69])

**Green Supply Chain Integration Intention (GSCII)**

GSCII1: The enterprise is willing to make efforts towards implementing green supply chain integration. (Jum'a et al.,2022[70])

GSCII2: The enterprise has formulated plans to implement green supply chain integration. (Jum'a et al.,2022[70])

GSCII3: Implementing green supply chain integration implies numerous benefits for the enterprise. (Singh and Joshi,2024[71])

GSCII4:The enterprise holds high expectations for the outcomes of implementing green supply chain integration. (Singh and Joshi,2024[71])

GSCII5: Green supply chain integration is an essential green strategy in the production and operation process of the enterprise. (Jum'a et al.,2022[70])
