# Supplementary figures and images for: Institutional pressures and green supply chain integration intention: Evidence from Chinese manufacturing firms
Source: PLoS One. 2025 May 7;20(5):e0322200. doi: 10.1371/journal.pone.0322200 (PMC12058135; doi:10.1371/journal.pone.0322200)

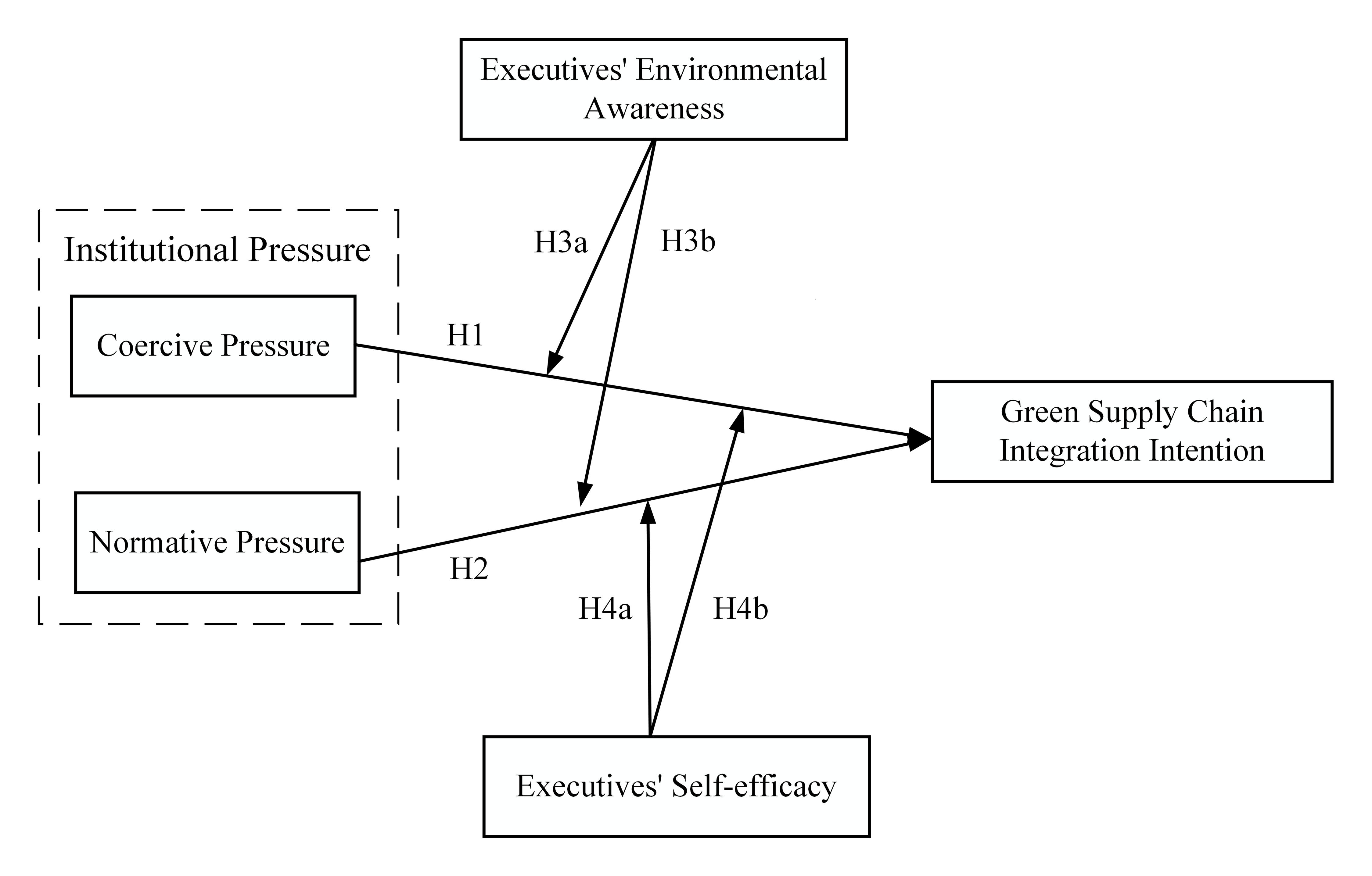

Supplement: S3 Fig1 — XXX. (TIF) [file pone.0322200.s003.tif]

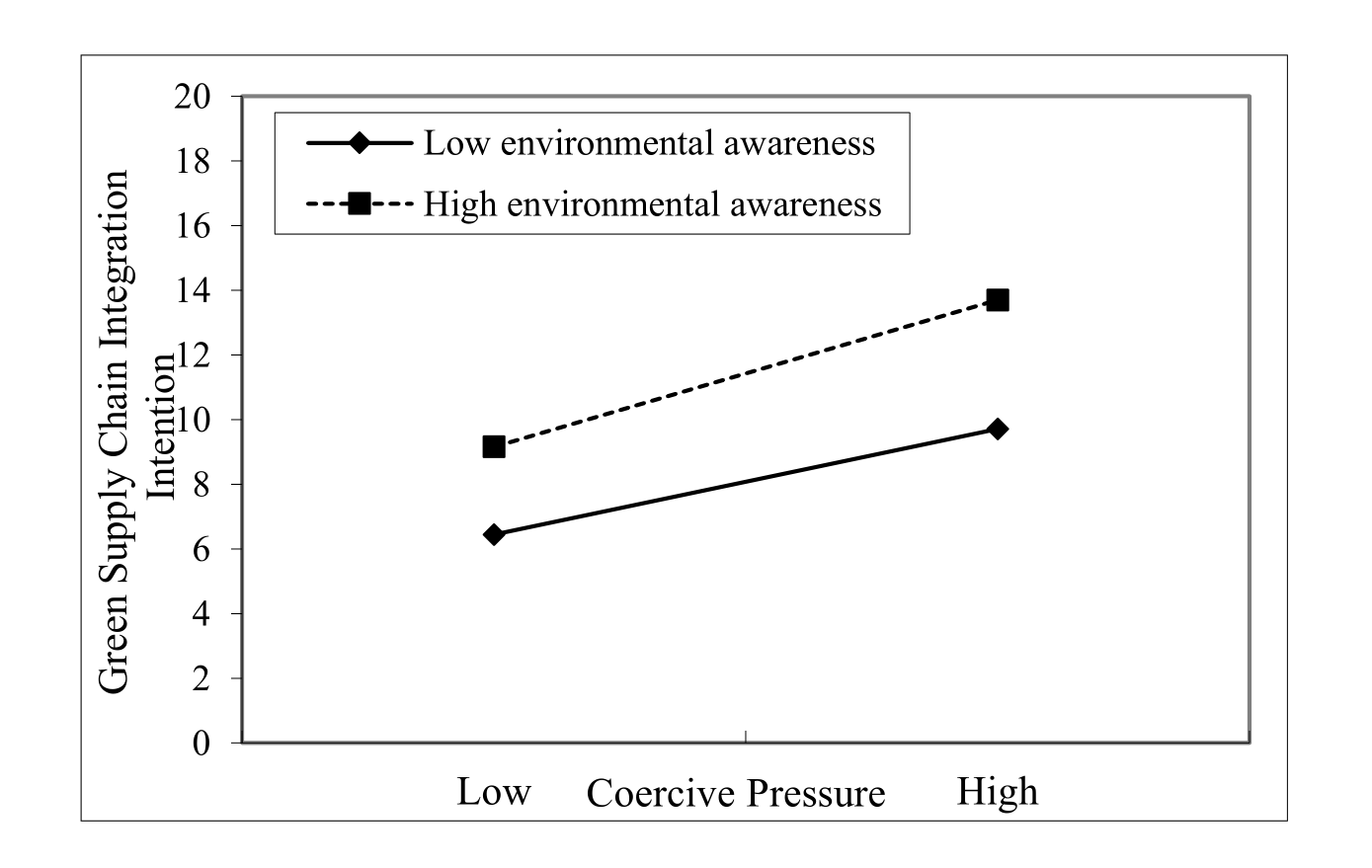

Supplement: S4 Fig2 — (TIF) [file pone.0322200.s004.tif]

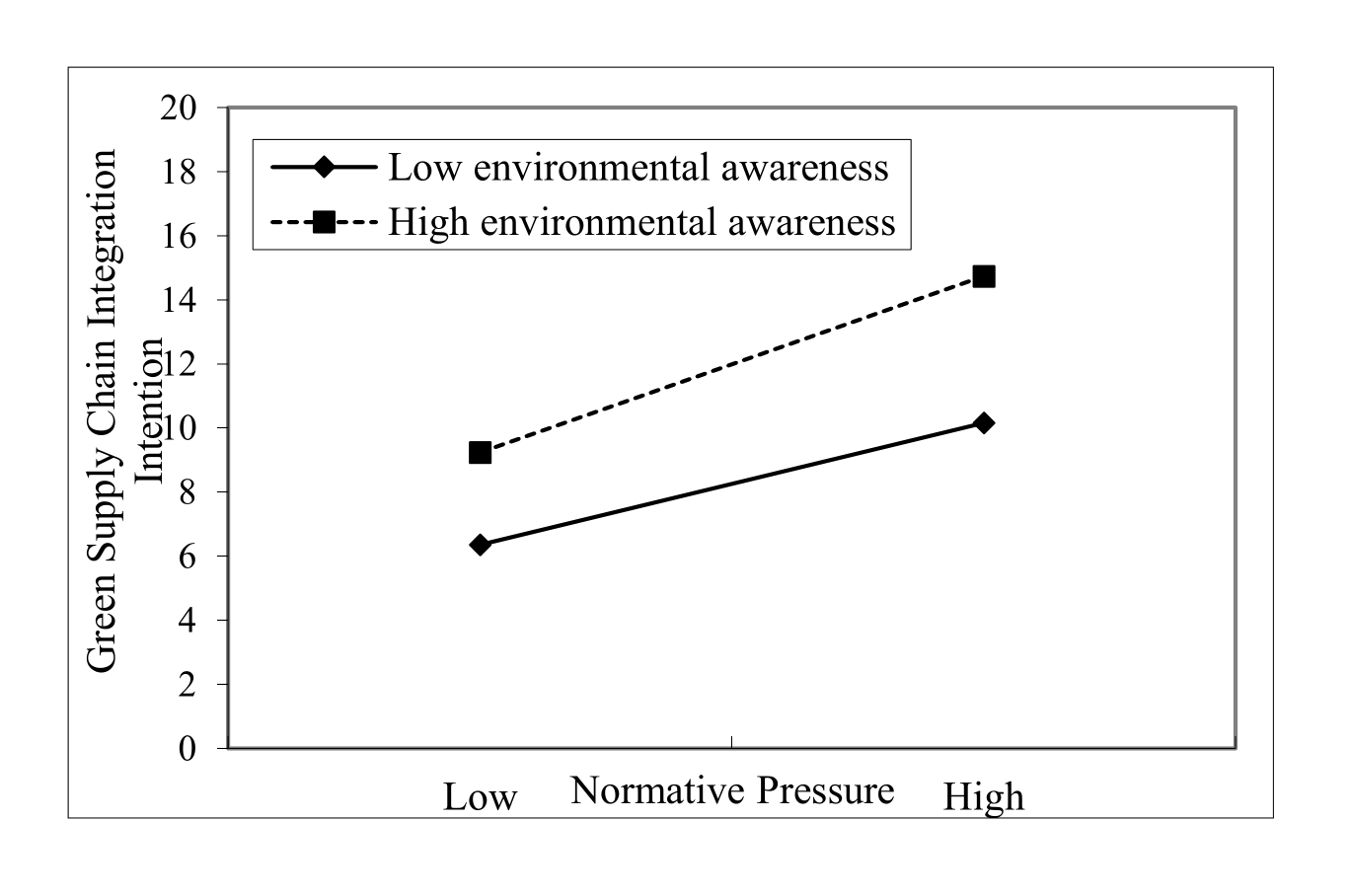

Supplement: S5 Fig3 — (TIF) [file pone.0322200.s005.tif]

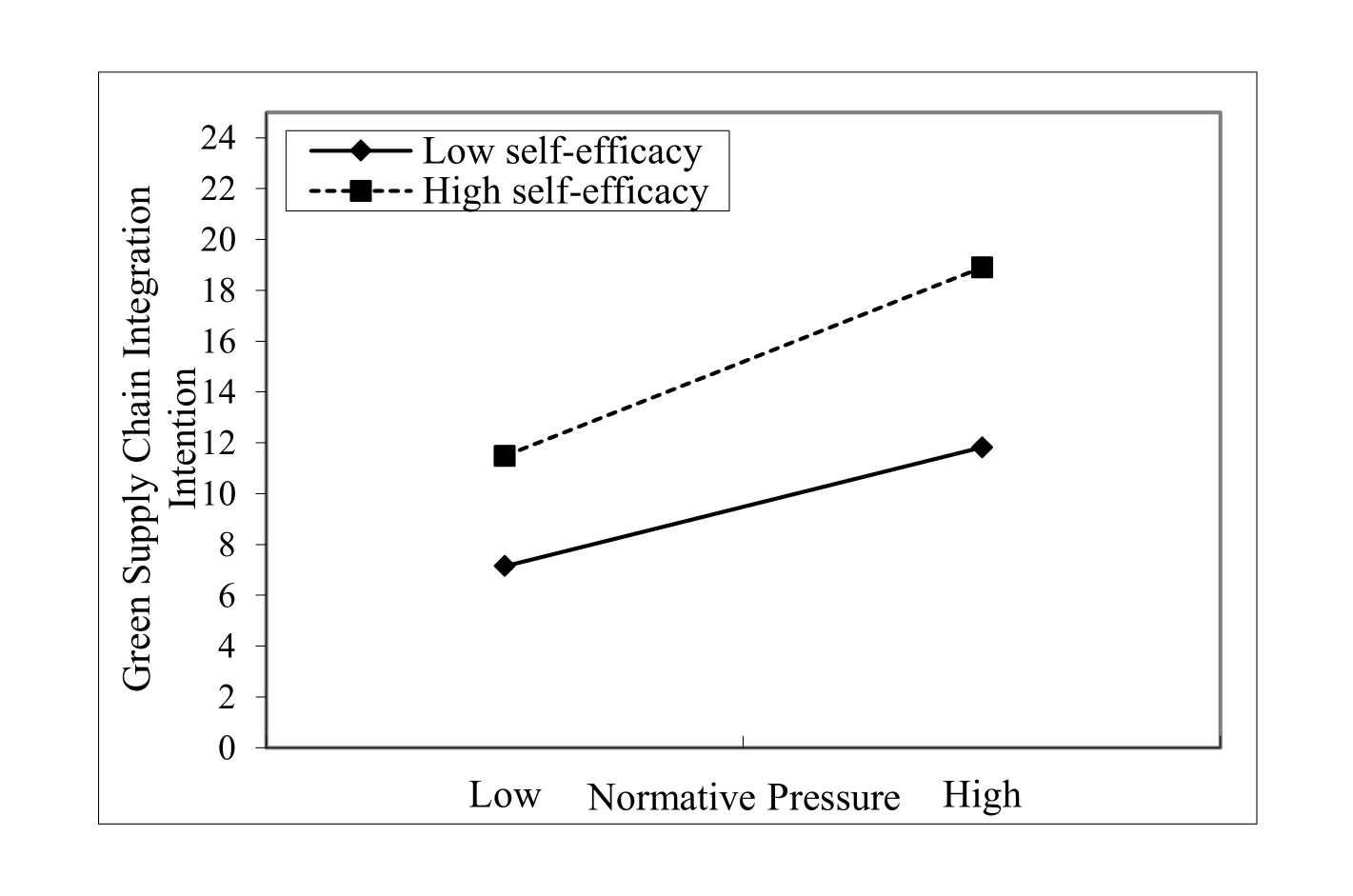

Supplement: S6 Fig4 — (TIF) [file pone.0322200.s006.tif]

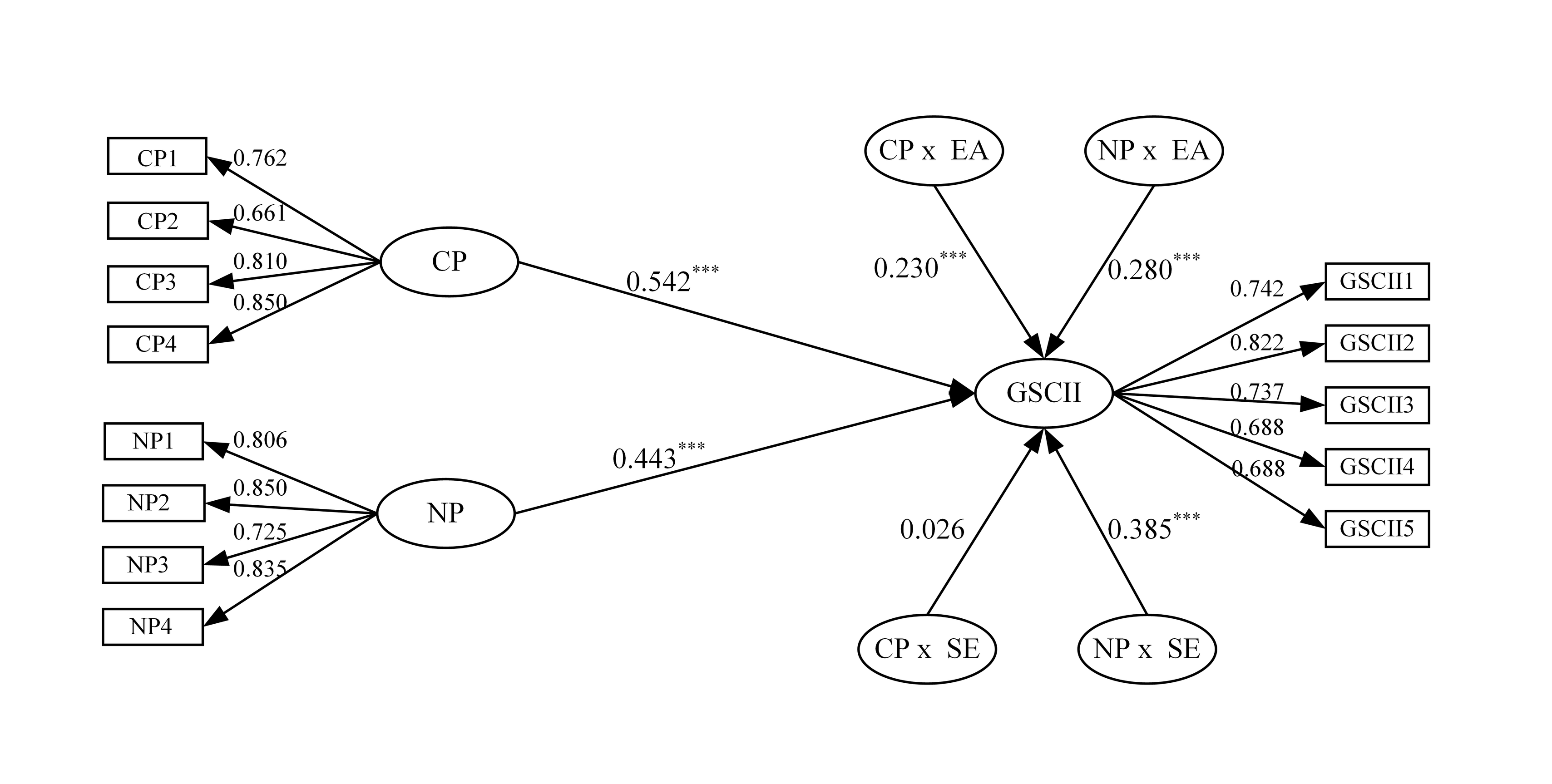

Supplement: S7 Fig5 — (TIF) [file pone.0322200.s007.tif]
